# Surgical suction filter-derived bone graft displays osteogenic miRNA and mRNA patterns

**DOI:** 10.1007/s00068-023-02350-5

**Published:** 2023-08-30

**Authors:** Rald V. M. Groven, Job T. Blokhuis, Martijn Poeze, Martijn van Griensven, Taco J. Blokhuis

**Affiliations:** 1 Department of Cell Biology-Inspired Tissue Engineering, MERLN Institute for Technology-Inspired Regenerative Medicine, Maastricht University, Maastricht, The Netherlands; 2 Division of Trauma Surgery, Department of Surgery, Maastricht University Medical Center, Maastricht, The Netherlands

**Keywords:** microRNAs, Bone grafting substitute, Graft extender, Non-union, Bone regeneration, Stem cells

## Abstract

**Purpose:**

Recently, a surgical suction filter device was introduced which aims at generating a suction filter-derived bone grafting substitute (SF-BGS). The osteogenic capacity of this grafting material, however, is unclear. MicroRNAs (miRNAs) and osteogenic mRNAs may influence these processes. The aim of this study was therefore to investigate the quality of the SF-BGS by determining the expression of miRNAs and osteogenic mRNAs.

**Methods:**

Samples were collected during non-union surgery. Upon exposure of the intramedullary canal, the surgical vacuum system was fitted with the suction filter device containing collagen complex and synthetic β-TCP: (Ca_3_(PO_4_)_2_, granule size 5–8 mm, total volume 10 mL (Cerasorb Foam^®^, Curasan AG, Kleinostheim, Germany). As a control, venous blood was used as in current clinical practice. Samples were snap-frozen and mechanically disrupted. MiRNAs and mRNAs were isolated, transcribed, and pooled for qPCR analysis. Lastly, mRNA targets were determined through in silico target analyses.

**Results:**

The study population consisted of seven patients with a posttraumatic long bone non-union (4♀; mean age 54 ± 16 years). From the array data, distinct differences in miRNA expression were found between the SF-BGS and control samples. Osteogenic marker genes were overall upregulated in the SF-BGS. Qiagen IPA software identified 1168 mRNA targets for 43 of the overall deregulated miRNAs.

**Conclusion:**

This study revealed distinctly deregulated and exclusively expressed osteogenic miRNAs in SF-BGS, as well as overall enhanced osteogenic marker gene expression, as compared to the venous blood control group. These expression profiles were not seen in control samples, indicating that the derived material displays an osteogenic profile. It may therefore be a promising tool to generate a BGS or graft extender when needed.

**Supplementary Information:**

The online version contains supplementary material available at 10.1007/s00068-023-02350-5.

## Introduction

A frequently applied, two-step surgical procedure in bone reconstruction of large defects is the Induced-Membrane-Technique (IMT) [1]. In the first step, debridement of the defect is followed by placement of a cement spacer. This spacer induces membrane formation in a physiological response to the foreign body. In the second step, the cement spacer is removed and the remaining cavity is filled with bone grafting material, for which autograft from the iliac crest is frequently used [2]. When treating larger bone defects, the restricted volume and osteogenic capacity of autograft are regarded as limiting factors [3]. Apart from that, the complications of harvesting autograft are also a drawback to its use [2]. To improve the results of autograft, techniques such as the Reamer-Irrigator-Aspirator (RIA) can be applied, aiming to enhance volume as well as the osteogenic capacity of the grafting material [2, 4]. The RIA procedure was first introduced to reduce the incidence of pulmonary fat embolisms in femur fracture patients, and although it is effective in harvesting larger quantities of autograft and yields more osteogenic cells than from the iliac crest, the procedure is invasive, costly, and bears the risk of significant blood loss and/or fracturing the healthy bone [5]. In addition, RIA is not always feasible, for example when an intramedullary nail is present. Alternative methods to harvest osteogenic material are therefore still needed.

During fracture surgery, significant amounts of bone marrow and bone tissue exit the surgical site following procedures such as drilling, reaming, or flushing. Under normal circumstances, this mixture is aspirated from the surgical field via the standard surgical vacuum system and discarded. Recently, a new suction filter device was introduced that can be connected to this standard surgical vacuum system. In this suction filter device, a scaffold material is placed, which is then loaded with aspiration material from the surgical site [6].

Work by Henze et al*.* has shown that stromal cells harvested with this surgical suction filter device were superior in proliferation as compared to those harvested via bone marrow aspiration, but more research is required to assess osteogenic capacities of the derived grafting material [7]. A first step is to examine cellular communication and signalling related to osteogenesis. This cellular communication and signalling occurs in part through the expression of specific microRNAs (miRNAs) and osteogenic messenger RNAs (mRNAs) [8].

MiRNAs are small, non-coding RNA molecules of roughly 22 nucleotides long. They post-transcriptionally regulate gene expression by binding, or cleaving target mRNAs, thereby modulating the proteome of a cell. Moreover, one miRNA targets different mRNAs, meaning that several cellular communication mechanisms are influenced simultaneously [9]. MiRNAs have been extensively researched in the field of bone regeneration and are known to be involved in healthy, as well as diseased fracture healing [8, 10].

This study therefore aims to investigate the quality of a novel surgical suction filter-derived bone grafting substitute (SF-BGS) by determining the expression of miRNAs and osteogenic mRNAs to assess its osteogenic profile.

## Materials and methods

### Human samples

Patients were consecutively included. Inclusion criteria were age ≥ 18 years and a posttraumatic, non-infected non-union of a long bone. Exclusion criteria were infected non-unions, patients undergoing bone marrow suppressive treatment, as well as pathological bone marrow and haematological conditions. Infection status was assessed by cultures, taken intraoperatively, which were cultured for 2 weeks.

**Fig. 1:**
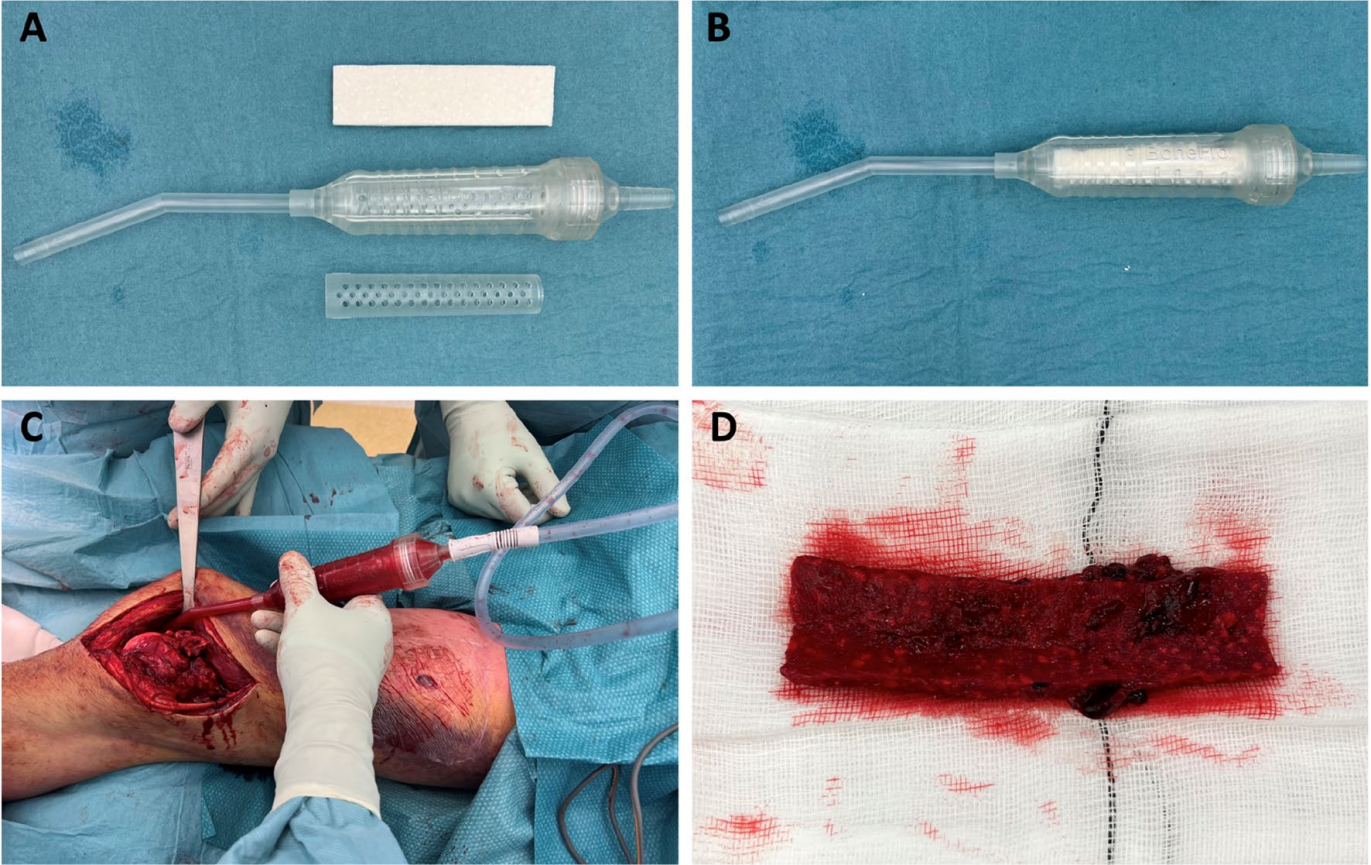
Intraoperative workflow of harvesting the suction filter-derived bone grafting substitute. The device (**A**) is loaded with the filter material (**B**): collagen complex and synthetic β-TCP: (Ca_3_(PO_4_)_2_, granule size 5–8 mm, total volume 10 mL (Cerasorb Foam®, Curasan AG, Kleinostheim, Germany). Harvesting was performed upon exposure of the intramedullary canal (**C**), and the harvested filter material (**D**) was then processed for further analyses. All procedures were performed by two senior consultants (MP & TB)

All samples were harvested during the second stage of the IMT. After removal of the cement spacer and the subsequent exposure of the intramedullary canal, the surgical vacuum system was fitted with the suction filter device (BoneFlo, TissueFlow GmbH, Essen, Germany) containing a filter that was loaded with collagen complex and synthetic β-TCP: (Ca_3_(PO_4_)_2_, granule size 5–8 mm, total volume 10 mL (Cerasorb Foam^®^, Curasan AG, Kleinostheim, Germany), throughout this manuscript further referred to as ‘’filter material’’. During subsequent suctioning of bone marrow from the exposed intramedullary canal, the filter material was saturated with aspirated bone marrow, blood, and debris from the affected bone’s intramedullary canal. Saturation of the filter material was visually confirmed. Samples from the derived graft, filter material with material from the surgical site, were processed as described below. All surgical procedures were performed by two senior consultants (MP & TB) (Fig. 1).

As a control, venous blood was used from healthy volunteers, since this is how the filter material is coated in clinical practice, and subsequently processed according to the same experimental workflow in the laboratory. This study was approved by the local ethical committee of the MUMC+ (approval number: METC 2022-3218). The study was performed according to the Declaration of Helsinki in its most recent version.

### Sample processing and RNA extraction

After saturation, the graft was cut into 1.5 ml fragments and transferred to 2 ml microcentrifuge tubes (Biotix, San Diego, USA) using sterile tweezers. The tubes were directly snap frozen in liquid nitrogen. To mimic the surgical circumstances most accurately, control samples were obtained by soaking the filter material in whole blood at room temperature for 20 min prior to snap freezing. All samples were disrupted by adding 1 ml of Trizol Reagent (Thermo Fisher Scientific, Waltham, USA) after which the homogenate was snap frozen and subsequently lysed for five minutes using a Qiagen TissueLyser LT (Qiagen, Venlo, The Netherlands).

RNA was extracted by chloroform phenol extraction using GlycoBlue co-precipitant (Thermo Fisher Scientific, Waltham, USA) according to the manufacturer’s instructions. The amount and purity of the RNA were determined by spectrophotometry using a CLARIOstar Plate Reader and LVis Plate Adapter (Isogen Life Science, De Meern, The Netherlands). For samples to be included in this study, RNA purity cut-off values for the A260/A230 and A260/A280 ratios were set at 1.7 and 1.8, respectively. RNA isolates were then stored at -80˚C until further analysis.

### Quantitative PCR miRNA arrays

To analyse miRNAs, 150 ng of template RNA were transcribed to cDNA using the miRCURY LNA RT kit (Qiagen, Venlo, The Netherlands) according to the manufacturer’s instructions.

The human fibrosis focus array (YAHS-217ZD-4, Qiagen, Venlo, The Netherlands) was selected and used. This array included miRNAs that have been shown to be involved in bone regeneration. Patient and control cDNA samples were pooled using equal amounts of cDNA from each sample. The 96-well panel contains 84 unique primers for individual miRNAs, four pre-defined housekeeper genes, and eight reverse transcription and PCR controls. MiRNA gene expression levels were determined via qPCR by the cycle number (Cq), using the CFX96 Real-Time PCR system (Bio-Rad, Munich, Germany). The quality of the qPCR array reactions was assessed through melting curve analyses, in combination with a cut-off Cq value of 35, above which miRNAs were not included in further analysis due to negligible or absent presence.

Gene expression was normalised by calculating a normalisation factor according to the geNorm normalisation method for accurate and reliable normalisation. Fold regulations of ≥ 2 or ≤ -2 were considered significantly deregulated and were included for further analyses. For miRNAs that only displayed expression in the SF-BGS group, gene expression was analysed by normalisation to the mean of six pre-defined reference genes and the ∆Cq method (Cq reference genes—Cq gene of interest). A miRNA with an absolute ∆Cq ≥ 2 was considered as deregulated.

### Quantitative PCR of osteogenic marker genes

To analyse mRNAs, 150 ng template RNA were transcribed to cDNA using the iScript cDNA synthesis kit (BioRad, California, USA) according to manufacturer’s instructions.

The following osteogenic marker genes were included for analyses: alkaline phosphatase (ALP), osterix (OSX), runt related transcription factor 2 (RUNX2), and osteopontin (OPN). Controls included reverse transcriptase and negative PCR controls. CPSF-6 was chosen as a housekeeper gene. The specific primers are listed in table 1. MRNA gene expression levels were determined via qPCR by the cycle number (Cq), using the CFX96 Real-Time PCR system (Bio-Rad, Munich, Germany). All cDNA samples of each individual patient were analysed in triplicates. The quality of the qPCR reactions was assessed as described in the previous paragraph. Data were analysed by means of the 2^−∆∆Cq^ method.

**Table 1:** Human quantitative PCR primers alkaline phosphatase (ALP), osterix (OSX), runt related transcription factor 2 (RUNX2), osteopontin (OPN), cleavage and polyadenylation specific factor 6 (CPSF6)

Gene	Forward primer 5′→3′	Reverse primer 5′ → 3′	Accession code
ALP	ACTGGTACTCAGACAACGAGAT	ACGTCAATGTCCCTGATGTTATG	NM_000478.4
OSX	CCTCTGCGGGACTCAACAAC	AGCCCATTAGTGCTTGTAAAGG	NM_001173467.3
RUNX2	TCAACGATCTGAGATTTGTGGG	GGGGAGGATTTGTGAAGACGG	NM_001024630.4
OPN	CTCCATTGACTCGAACGACTC	CGTCTGTAGCATCAGGGTACTG	NM_000582
CPSF6	AAGATTGCCTTCATGGAATTGAG	TCGTGATCTACTATGGTCCCTCTCT	NM_007007.3

### In silico miRNA target prediction

To determine the mRNA targets of each of the significantly deregulated miRNAs, Qiagen’s Ingenuity Pathway Analysis (IPA) (Qiagen, Venlo, The Netherlands) software was used. For this study, the following cell types were included in the analysis: mesenchymal stem cells (MSCs), neutrophils, chondrocytes, osteoblasts, stromal cells, fibroblasts, and bone marrow cells. Only experimentally observed and highly predicted human miRNA targets were incorporated in the analyses.

## Results

The study population consisted of seven patients with a long bone non-union (4♀; mean age 54 ± 16 years). Five samples were obtained from a non-union of the tibia, one from the femur, and one from the radius.

### Identification of differentially expressed miRNAs: quantitative PCR arrays

**Fig. 2:**
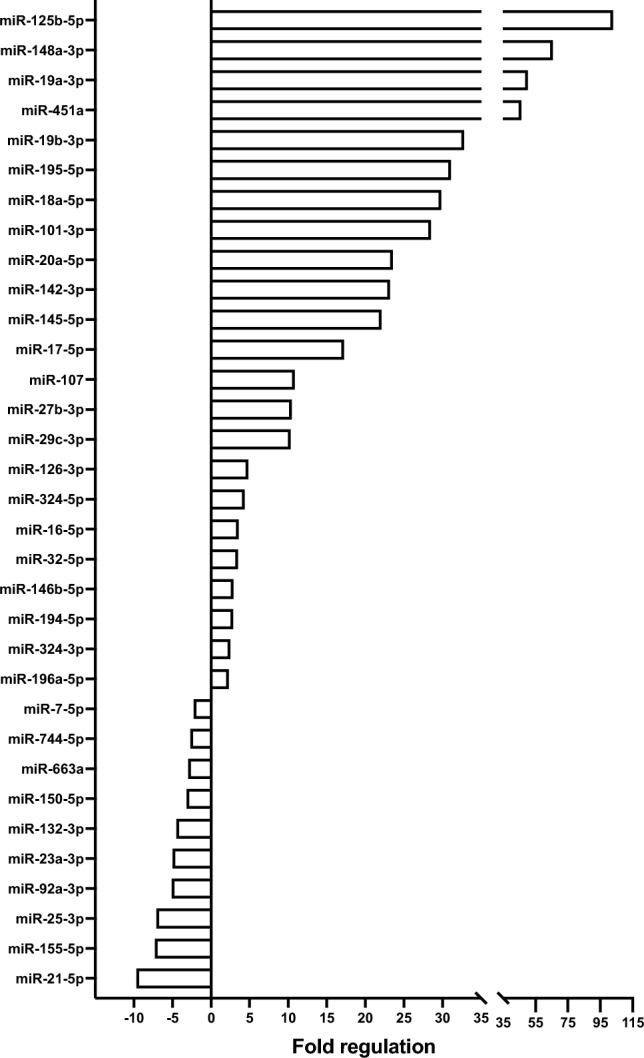
Deregulated microRNAs from the pooled sample group (*n* = 7) in Qiagen's fibrosis array. Results are normalised to the mean of six pre-defined housekeeper genes, whole blood from healthy volunteers (*n* = 5) was used as a control. All shown miRNAs displayed a fold regulation of ≥ 2 or ≤ – 2

**Fig. 3:**
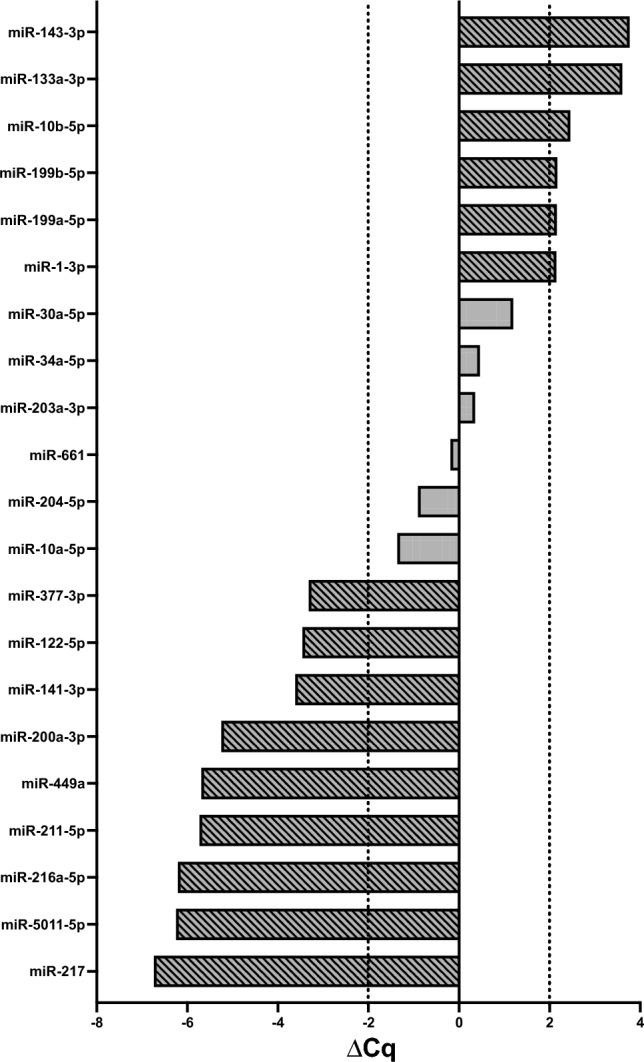
Specific microRNAs that were only expression in the SF-BGS sample group (*n* = 7). Qiagen’s fibrosis array was used, results are normalised to the mean of six pre-defined housekeeper genes. Black dotted lines and a diagonal fill pattern display that the miRNA was considered deregulated, showing an absolute ∆Cq ≥ 2. Positive ∆Cq values depict upregulation, negative ∆Cq values depict downregulation

**Fig. 4:**
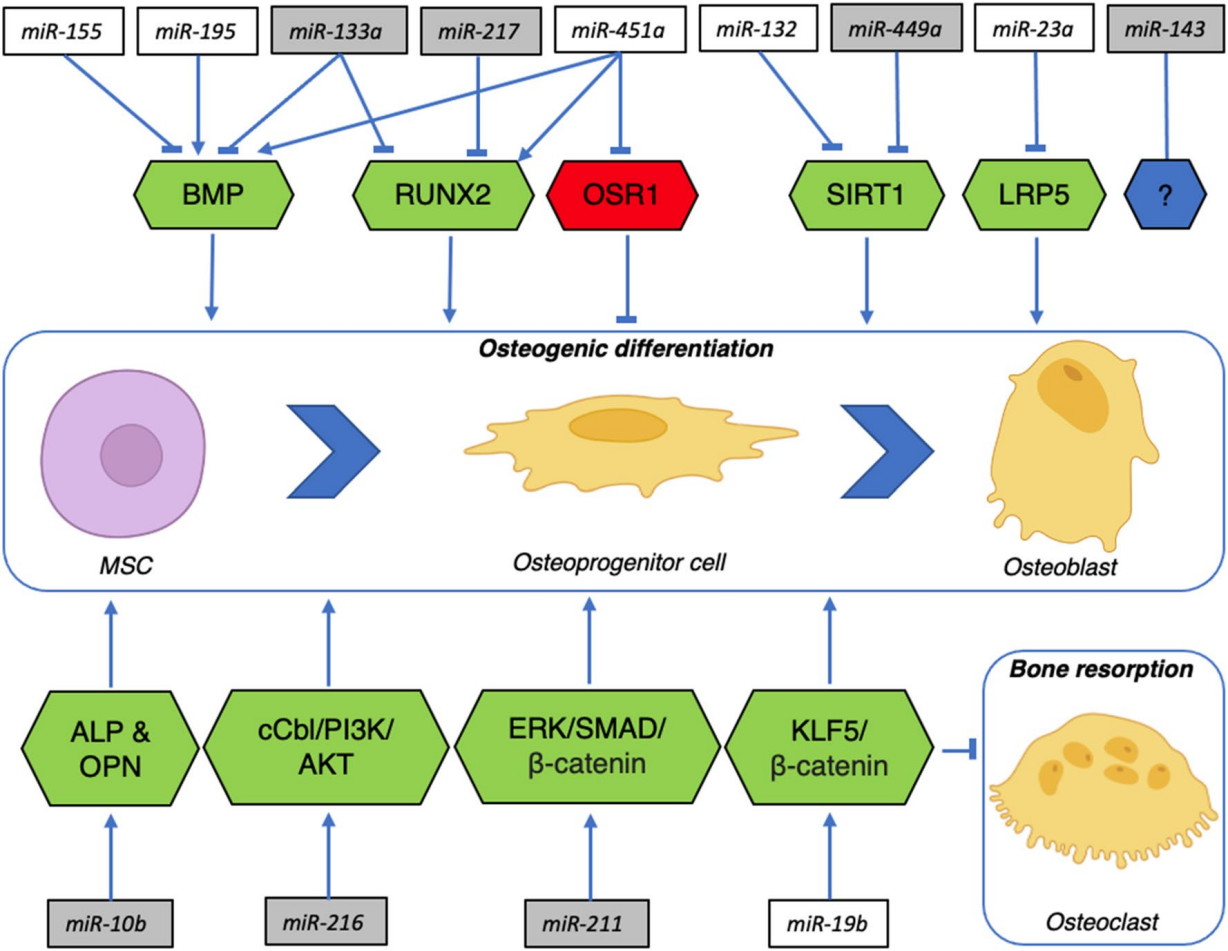
Schematic overview of the role of the most deregulated miRNAs in the suction filter-derived bone grafting substitute (SF-BGS). MiRNAs that are exclusively expressed in SF-BGS are depicted in grey boxes, deregulated miRNAs in SF-BGS as compared to the control group are depicted in white boxes. Green boxes depict gene targets with a stimulatory effect on osteogenic differentiation, red boxes depict targets with an inhibitory effect on osteogenic differentiation. Mesenchymal stem cell (MSC), Bone Morphogenic Protein (BMP), Runt related transcription factor 2 (RUNX2), Odd Skipped Related 1 (OSR1), Sirtuin 1 (SIRT 1), LDL receptor related protein 5 (LRP5), Alkaline Phosphatase (ALP), Osteopontin (OPN), Casitas B-lineage lymphoma (cCBL), Phosphatidylinositol-3-kinase (PI3K), Protein kinase B (AKT), Extracellular signal-Regulated Kinases (ERK), Suppressor of Mothers against Decapentaplegic (SMAD), Krüppel‐like factor 5 (KLF5)

From the 84 miRNAs that were analysed in the fibrosis array, 77 miRNAs were detected in the SF-BGS group, as compared to 56 miRNAs in the control group (Figs. S1 and S2). In the SF-BGS group, 23 and 10 miRNAs were significantly up and downregulated, respectively, as compared to the control group (Fig. 2). Twenty-one miRNAs only showed expression in the SF-BGS group, of which 15 miRNAs were considerably deregulated with an absolute ∆Cq ≥ 2. From these 15, nine were downregulated and six upregulated (Fig. 3). The potential impact on osteogenic differentiation of the most deregulated miRNAs as described in literature is shown in Fig. 4.

### Osteogenic marker gene expression

**Fig. 5:**
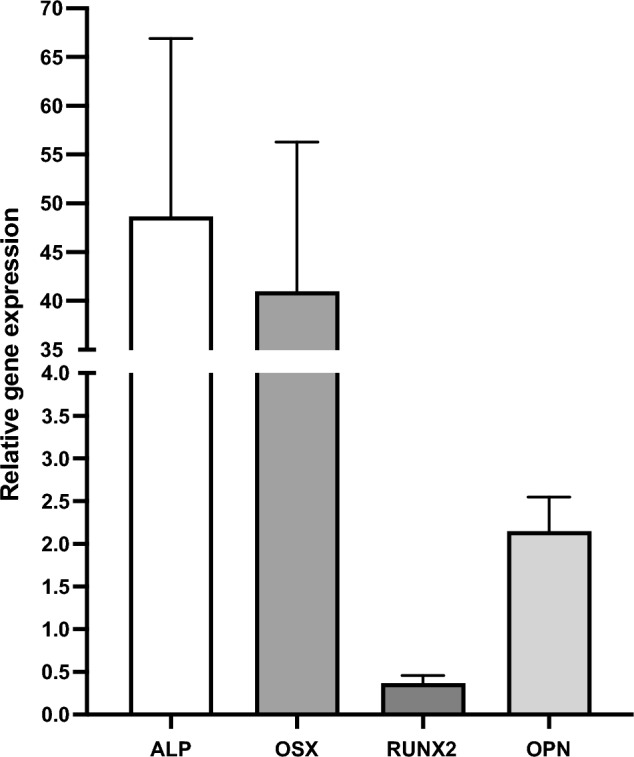
Mean relative gene expression alkaline phosphatase (ALP), osterix (OSX), runt related transcription factor 2 (RUNX2), and osteopontin (OPN) in surgical suction filter-derived bone grafting substitute

All samples in the SF-BGS group showed increased relative gene expression of the examined osteogenic marker genes (Fig. 5). Most prominent upregulations were observed for ALP and OSX, showing a 49- and 41-fold increase in relative gene expression, respectively. This upregulation was less evident for OPN, showing a twofold increase in relative gene expression. RUNX2 was downregulated as compared to the housekeeper genes. None of the control samples displayed osteogenic marker gene expression, which led us to perform data analyses through the 2^−∆Cq^ method instead of the 2^−∆∆Cq^ method.

### In silico target analysis

**Fig. 6:**
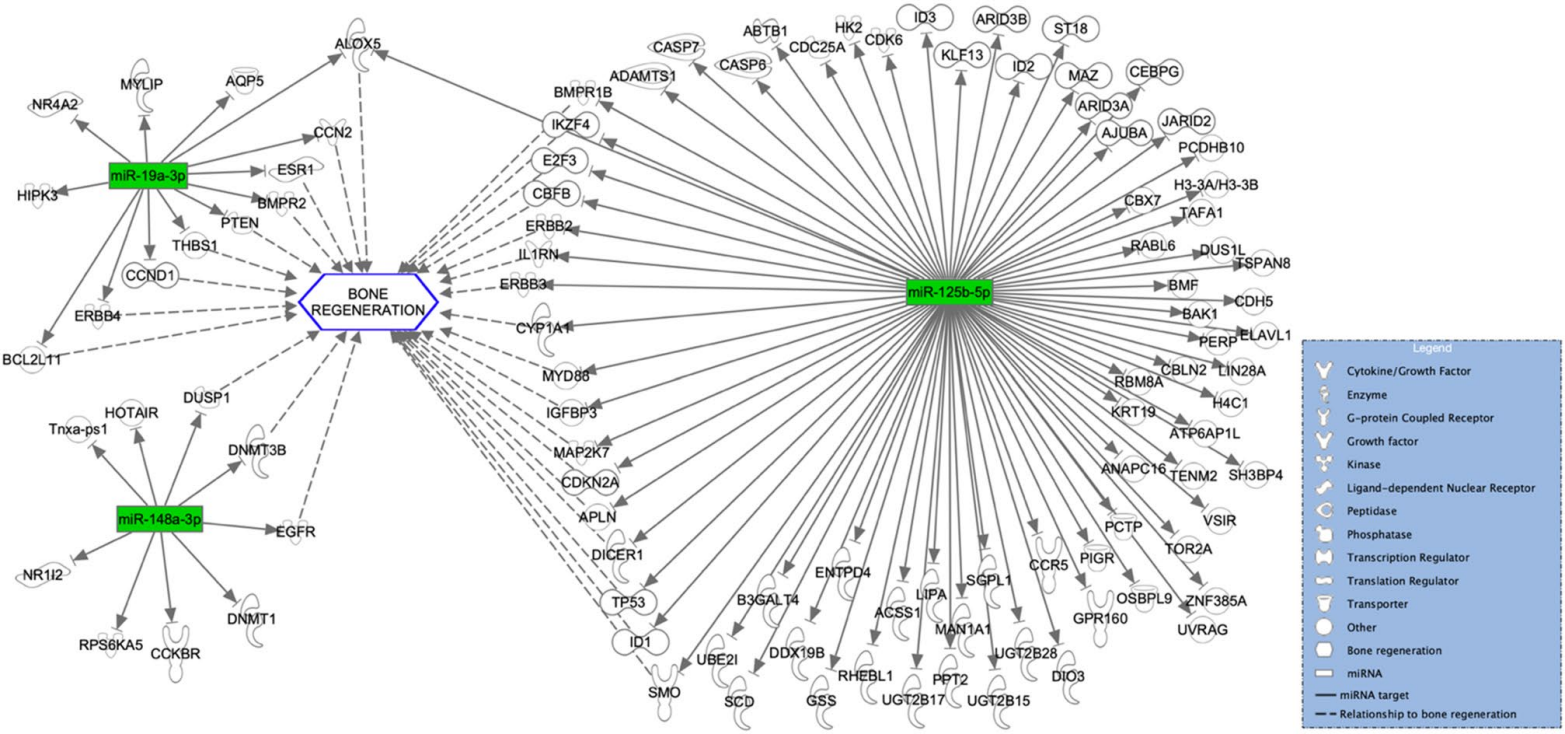
In silico target analysis, performed with Qiagen Ingenuity Pathway Analysis (IPA) software, of 3 miRNAs whose expression was upregulated in the SF-BGS group as compared to the control group. Green colour represents upregulated miRNA expression, dotted arrows indicate a relationship to bone regeneration

**Fig. 7:**
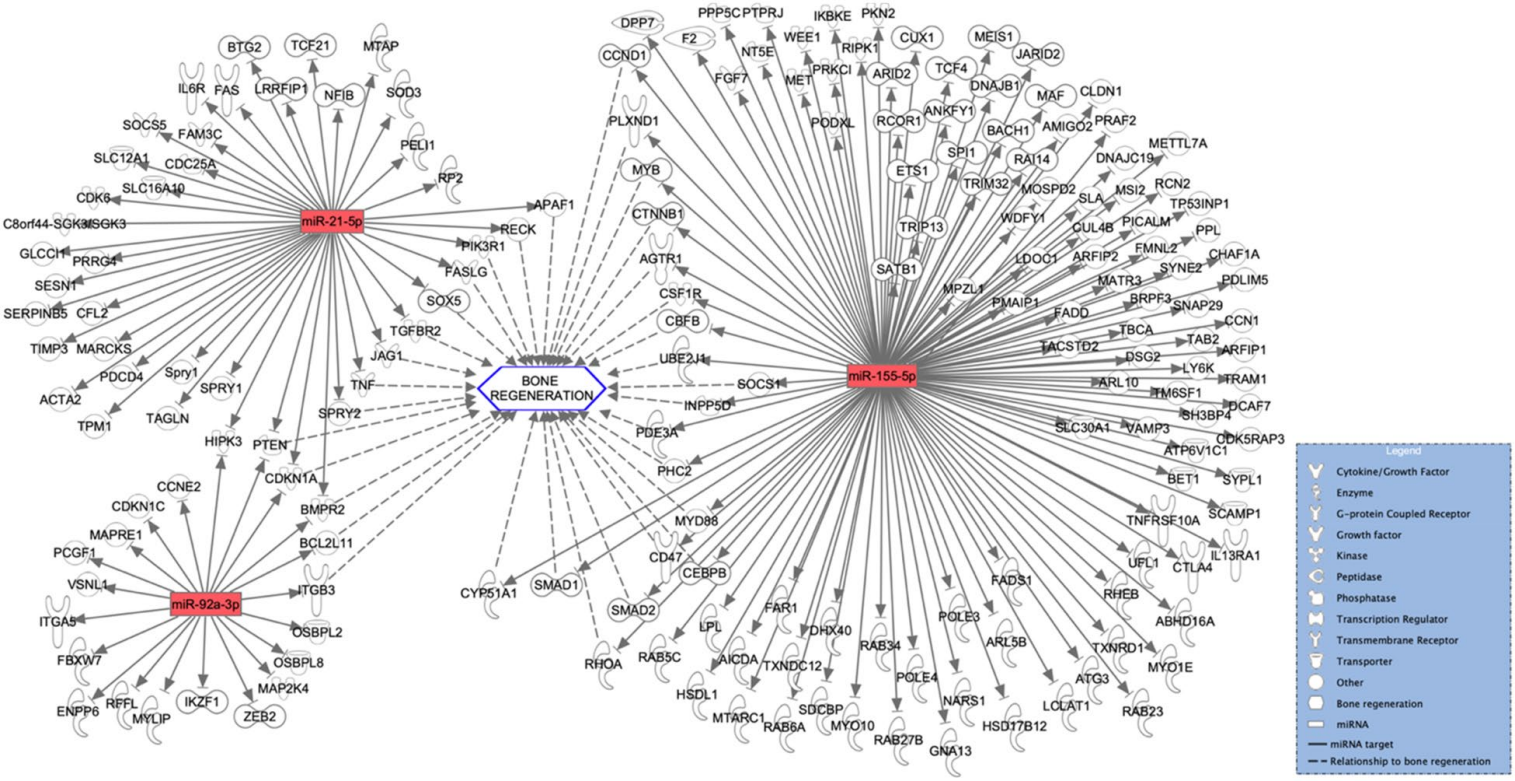
In silico target analysis, performed with Qiagen Ingenuity Pathway Analysis (IPA) software, of 3 miRNAs whose expression was downregulated in the SF-BGS group as compared to the control group. Red colour represents downregulated miRNA expression, dotted arrows indicate a relationship to bone regeneration

**Fig. 8:**
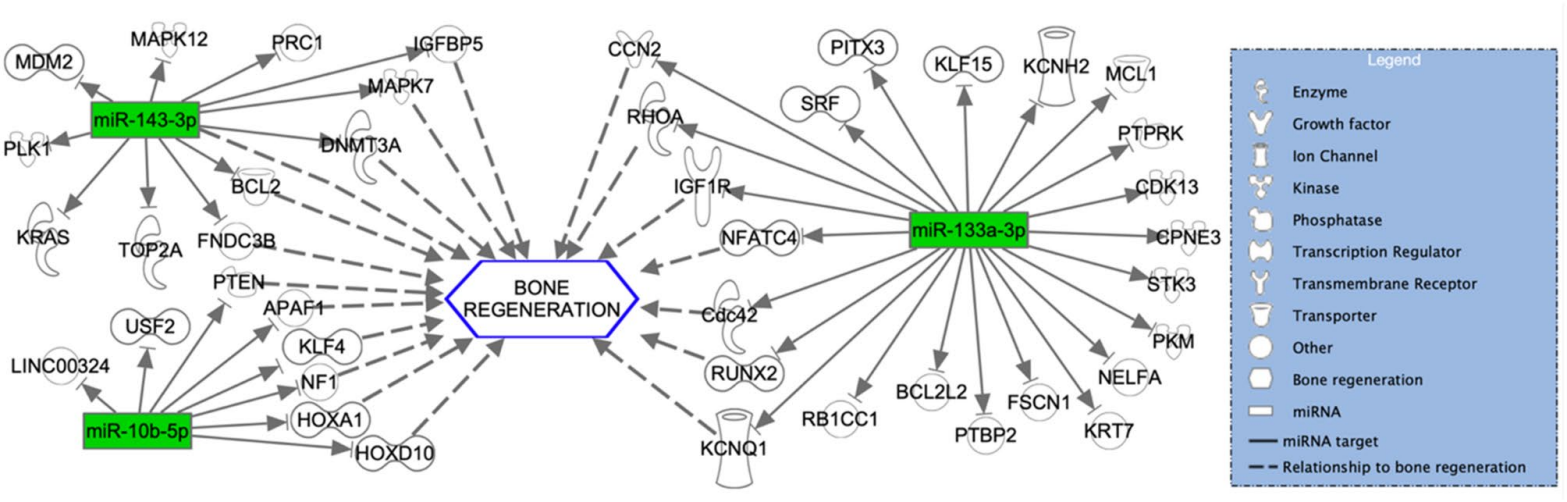
In silico target analysis, performed with Qiagen Ingenuity Pathway Analysis (IPA) software, of 3 miRNAs which were exclusively expressed in the SF-BGS group. Green colour represents upregulated miRNA expression as compared to the housekeeper genes, dotted arrows indicate a relationship to bone regeneration

**Fig. 9:**
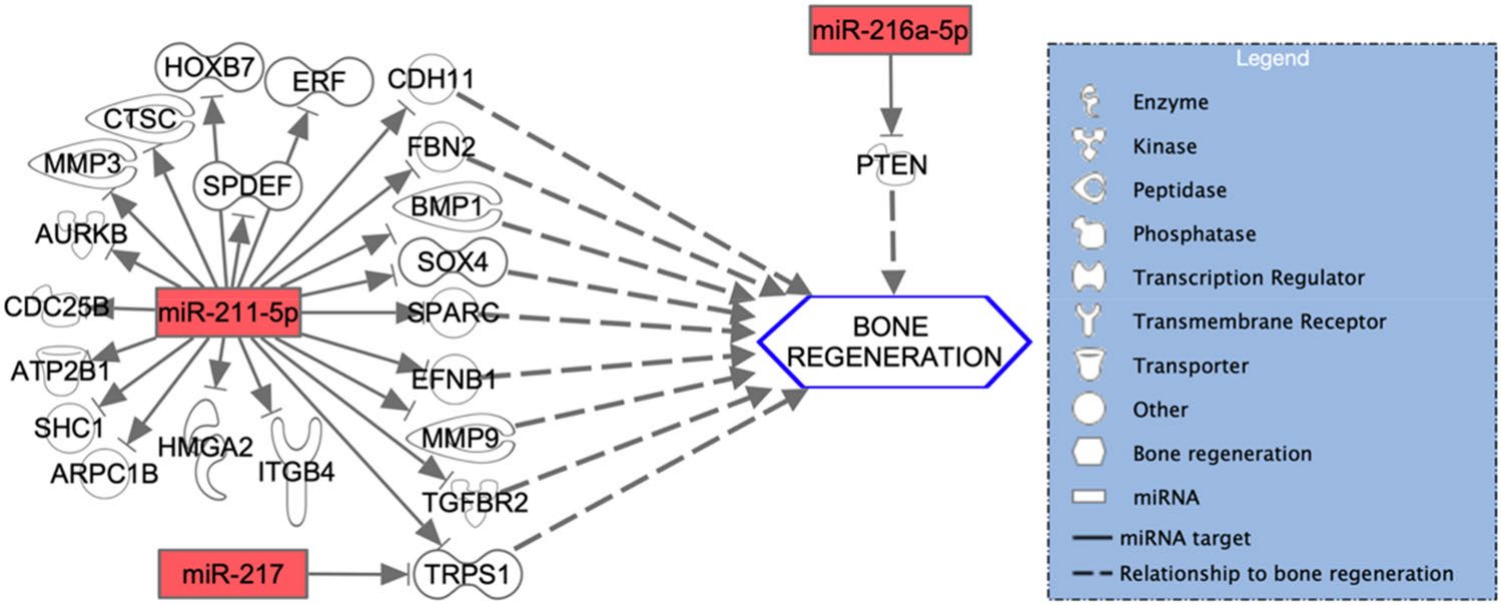
In silico target analysis, performed with Qiagen Ingenuity Pathway Analysis (IPA) software, of 3 miRNAs which were exclusively expressed in the SF-BGS group. Red colour represents downregulated miRNA expression as compared to the housekeeper genes, dotted arrows indicate a relationship to bone regeneration

Qiagen IPA software identified 1168 experimentally observed targets for 43 of the overall deregulated miRNAs. The miRNA-mRNA interactions of the three most down and upregulated miRNAs are depicted in Figs. 6 and 7. Furthermore, the miRNA-mRNA interactions of the three most down and upregulated miRNAs that were exclusively expressed in the SF-BGS are depicted in Figs. 8 and 9. Targets were involved in various aspects of bone regeneration including the inflammatory response. Among others, mRNAs involved in extravasation of immune cells, hypoxia, fibrosis, and angiogenesis were identified as targets of the selected miRNAs. Several miRNAs that were only expressed in the SF-BGS group showed involvements in osteogenesis, influencing mineralisation, collagen synthesis, calcium mobilisation, and angiogenesis. The in silico target prediction showed that several mRNA targets overlapped between the selected miRNAs, such as Phosphatase and Tensin homolog, Bone Morphogenic Protein Receptor 2, and Homeodomain Interacting Protein Kinase 3.

## Discussion

This study examined osteogenic gene expression in a novel technique to provide surgical suction derived BGS by determining the expression profiles of osteogenic miRNAs and mRNAs. The increased expression of several osteogenic miRNAs, as well as the increased early osteogenic marker expression in the SF-BGS, indicate that it may provide a bone grafting substitute that has suitable osteogenic capacities. Apart from offering an alternative to existing bone grafting materials, this novel device could also serve as a graft extender when needed.

### MiRNA expression compared to the control group

Among the miRNAs that were most upregulated compared to the control group were miR-195, miR-451a, and miR-19b. MiRNA-195 has shown to attenuate osteoporotic bone loss in vivo by activating Bone Morphogenic Protein (BMP)-2 mediated signalling pathways [11, 12]. Similarly, miR-451a enhances osteoblastogenesis and matrix mineralisation in vitro and in vivo by enhancing the expression of RUNX2 and BMP-4, whilst simultaneously suppressing Odd Skipped Related 1 [13]. MiR-19b promotes the osteogenic differentiation of MSCs in vitro and enhances fracture healing in vivo by activating KLF5/β-catenin signalling, which has also shown to decrease bone resorption [14]. Furthermore, the incorporation of miR-19b in scaffolding material has shown positive effects in the treatment of bone defects in rats [15]. Upregulation of these miRNAs in the SF-BGS thus promotes osteogenesis by influencing bone formation in several ways, such as osteoblastogenesis, mineralisation, and reduced bone resorption.

Among the miRNAs that were most evidently downregulated compared to the control group were miR-155, miR-23a, and miR-132. MiR-155 is overexpressed in various inflammatory diseases and cancers and restrains osteogenic differentiation by downregulating BMP signalling [16]. MiR-23a is known to inhibit osteogenic differentiation of human Bone Marrow MSCs by targeting the LDL receptor related protein 5 [17]. Lastly, miR-132 expression is increased in mechanical unloading, indicating a negative effect on osteogenic differentiation. Indeed, silencing miR-132 attenuated the negative effects of mechanical unloading [18]. Furthermore, miR-132 has proven to reduce osteogenic differentiation by directly targeting Sirtuin 1 (SIRT1), a key molecule in osteogenic differentiation [19]. The strong downregulations of these three miRNAs in the SF-BGS are therefore desirable, looking at the negative effects of these miRNAs in relation to bone regeneration. Furthermore, mechanically sensitive expression of specific miRNAs that reduce osteogenesis, such as miR-132, offers novel insights into cellular mechanisms that may underlie the effectiveness of permissive weight bearing in trauma patients [20].

Some deregulated miRNAs influence bone regenerative processes in an ambivalent manner. Among these was miR-21, most downregulated as compared to the control samples, which under physiological circumstances enhances osteogenic differentiation and matrix mineralisation, whilst simultaneously promoting bone resorption through enhanced osteoclastogenesis [21, 22]. Similarly, miR-92a, again downregulated as compared to the control group, is known to enhance osteoblast differentiation in vitro, but simultaneously inhibits angiogenesis, another key component of successful bone regeneration [23, 24]. Lastly, miRNA-148a has shown to enhance, as well as decrease osteoblastic differentiation. It facilitated osteogenic differentiation by targeting Dickkopf-related protein 1, a key protein in the WNT signalling pathway, thereby activating WNT signalling [25]. Additionally, it targeted Suppressor of Mothers Against Decapentaplegic (SMAD) Specific E3 Ubiquitin Protein Ligase 1, an upstream inhibitor of SMAD7/B-Cell Lymphoma 2 signalling, thereby having an osteoprotective function [26]. Apart from that, its close family member, miR-148b, promotes osteogenic differentiation and is currently under research for its potential application in biomaterials for bone reconstruction [27]. Contradictory, in vitro work by Liu et al*.* showed that miR-148a decreased osteoblastic differentiation [28]. An important feature to keep in mind when interpreting these data is that miRNAs have shown to elicit dose-dependent effects in vivo, being able to produce adverse effects depending on the applied dosage [29]. Apart from that, although the exact role of some of the identified miRNAs is still uncertain, their deregulation in comparison to the control samples is clear, necessitating further research.

### Exclusively expressed miRNAs in SF-BGS

Several miRNAs were expressed exclusively in the SF-BGS. Among the most upregulated miRNAs were miR-10b and miR-143. Enhanced expression of miR-10b has shown to promote osteogenic differentiation and bone formation both in vitro and in vivo, by enhancing the expression of ALP and OPN [30]. This matches the findings from this study since each of these two osteogenic marker genes resulted upregulated, combined with upregulated miR-10b expression in the SF-BGS group. Apart from that, although predominantly studied in the field of oncology, miR-10b is known to be a potent inducer of angiogenesis, a key component in bone formation [31]. MiR-143 was most upregulated of all miRNAs that were exclusively expressed in the SF-BGS. Although there is little research on miR-143 in the field of bone regeneration, its upregulation has shown to promote both angiogenesis as well as osteoblastic differentiation in an in vitro model, using murine MSCs and MC3T3-E1 cells [32]. Contrarily, Zhang et al*.* also showed that downregulating miR-143 promoted osteogenic differentiation in human Adipose-derived MSCs [33]. These studies show that this miRNA interacts with two key processes in bone regeneration: angiogenesis and osteogenic differentiation. miR-449a is downregulated in the SF-BGS and directly targets SIRT1. Under physiological circumstances, SIRT1 inhibits Nuclear Factor Kappa Beta signalling, thereby enhancing osteogenic differentiation whilst simultaneously reducing osteoclastogenesis [34, 35]. The downregulation of this miRNA should therefore be beneficial for bone formation. Looking at these miRNAs in relation to osteogenesis, it is again shown that osteogenesis is stimulated through several different pathways, such as osteoblastogenesis, apoptotic inhibition of chondrocytes, and mobilisation and proliferation of osteoprogenitor cells.

Several miRNAs that were exclusively detected in the SF-BGS had seemingly counter intuitive expression patterns. Some were not strongly upregulated but did show expression exclusively in the SF-BGS group, meaning that their presence elicits effects as compared to the control group. Among these were miR-211, miR-216, miR-217, and miR-133a. In vivo work by Wang et al*.* showed that overexpression of miR-211 greatly enhanced osteogenic differentiation by initiation of the Extracellular signal-Regulated Kinases/SMAD/β-catenin signalling cascade [36]. MiRNAs can also operate synergistically. This is shown by the fact that combined expression of miR-211 together with miR-204 has shown to reduce RUNX2 expression, thereby potentially reducing osteogenic differentiation. However, to date, such effects have only been observed in association with simultaneous miR-211 and miR-204 expression [37]. Research by Li et al*.* revealed that miR-216a enhances osteoblastogenesis and bone formation in vivo through a phosphatidylinositol-3-kinase/AKT mediated mechanism [38]. MiR-216a is also released by BMSCs upon hypoxia, after which it promoted proliferation, migration, and apoptotic inhibition of chondrocytes [39]. Placing these data in the perspective of fracture healing, it can be expected that the hypoxia induced release of miR-216a fits within the first stage of fracture healing, since disturbed vascularisation makes the fracture hematoma and fracture site a hypoxic environment [40]. The subsequent chondrogenesis is important for the next fracture healing phase considering endochondral ossification. MiR-217, downregulated in SF-BGS, has shown to both promote and inhibit bone regeneration. MiR-217 inhibits osteogenic differentiation by direct targeting of RUNX2 [41]. This matches the findings from this study, since miR-217 was indeed expressed exclusively in the SF-BGS and RUNX2 resulted slightly downregulated among the osteogenic marker genes. Contrarily it also enhanced osteogenic differentiation through activation of the WNT signalling pathway, by directly targeting Dickkopf-related protein 1 [42]. Lastly, upregulated in SF-BGS, overexpression of miR-133a targeted RUNX2 and BMP2 signalling, eliciting anti-angiogenic effects whilst also blocking the expression of osteogenic marker genes, matching the observed downregulation of RUNX2 [43]. Increased expression of this miRNA therefore seems disadvantageous for bone formation at first sight, but might also offer necessary negative feedback to prevent overactivation of osteogenic processes. These deregulations point out the requirement for more research into miRNA profiles in impaired fracture healing to establish whether the expression of one single miRNA can determine the patients’ outcome, or rather the integral miRNA transcriptome at the site.

### Osteogenic marker gene expression

No osteogenic marker gene expression was observed in control samples. This is in line with other studies that have described suboptimal results from autologous blood coagulum as a stand-alone bone grafting material [44]. In particular, a great increase in relative gene expression was observed for the early osteogenic differentiation markers ALP and OSX in SF-BGS. The well-known early osteogenic marker ALP, an enzyme which is increasingly expressed on the cell membrane of osteoprogenitor cells, provides the free phosphate that is required for the mineralisation of osteoid [45]. OSX also resulted strongly upregulated in the SF-BGS, showing a strong required stimulus for the commitment of osteogenic progenitor cells to preosteoblasts [46]. The increased expression of ALP and OSX in the SF-BGS therefore resembles a potentially osteogenic environment for osteoprogenitor cells. Although slight, RUNX2 resulted downregulated compared to all other osteogenic marker genes. This should be interpreted in relation to the fact that this study focusses on non-union patients, for which this surgical tool is in part developed, meaning that inadequate RUNX2 expression might be part of transcriptomal dysfunction in impaired fracture healing [47].

Since this is a pilot study, a limitation is its small patient population. This makes it impossible to correlate the identified osteogenic gene profiles to other patient demographics. However, the comparison with whole blood identified a clear osteogenic profile, which is an important step in the assessment of the future clinical value of the SF-BGS.

## Conclusions

This study has shown that a novel surgical vacuum filter device, used in bone reconstructive surgery, yields a graft material with a clear osteogenic profile. Distinctly deregulated, as well as exclusively expressed miRNAs, were identified in the SF-BGS, yielding an overall osteogenic miRNA signature, whilst also offering insights into bone regenerative cellular mechanisms, which are potentially miRNA chaperoned. In silico mRNA target analysis of this miRNA signature revealed a great number of targets, all of which were associated with key processes for successful bone regeneration, such as angiogenesis, matrix mineralisation, osteoblastogenesis, and cellular proliferation and migration.

Furthermore, this study revealed increased osteogenic marker gene expression in the SF-BGS. It may therefore be a promising surgical tool to generate a BGS, or function as a graft extender when needed. Further research should focus on the comparison between the osteogenic capacities of this SF-BGS and those of the current gold standards for bone grafting procedures, such as the iliac crest bone graft or the RIA procedure.

### Supplementary Information

Below is the link to the electronic supplementary material.

Supplementary file1 (PDF 173 KB)

## Data Availability

Data can be provided upon reasonable request.
