# Supplementary material for: Surgical suction filter-derived bone graft displays osteogenic miRNA and mRNA patterns
Source: Eur J Trauma Emerg Surg. 2023 Aug 30;50(1):315–26. doi: 10.1007/s00068-023-02350-5 (PMC10923964; doi:10.1007/s00068-023-02350-5)
Supplement: Supplementary file 1 — Supplementary file1 (PDF 173 KB) [file 68_2023_2350_MOESM1_ESM.pdf]

Supplementary Information

Surgical suction filter derived bone graft displays osteogenic miRNA and mRNA patterns

European Journal of Trauma and Emergency Surgery

Rald VM Groven[1,2*], Job T Blokhuis[1,2], Martijn Poeze[2], Martijn van Griensven[1], Taco J Blokhuis[2]

1 Department of Cell Biology-Inspired Tissue Engineering, MERLN Institute for Technology-Inspired Regenerative Medicine, Maastricht University, Maastricht, The Netherlands
2 Division of Trauma Surgery, Department of Surgery, Maastricht University Medical Center+, Maastricht, The Netherlands

* Correspondence: r.groven@maastrichtuniversity.nl

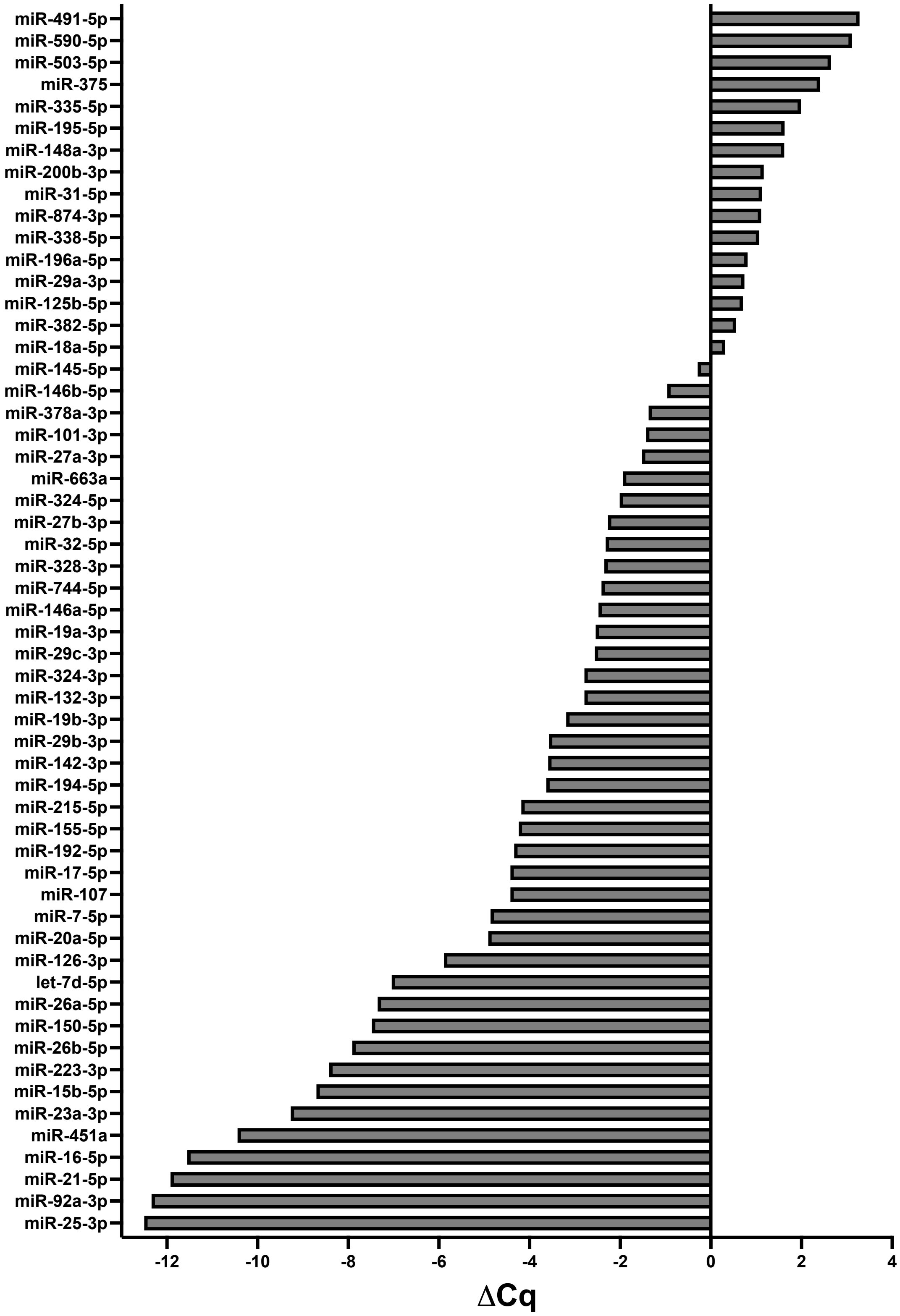

Figure S1. MicroRNA expression profile of the pooled control samples (n=5) in Qiagen's fibrosis array. Results are normalized to the mean of six pre-defined housekeeper genes and displayed as ΔCq.

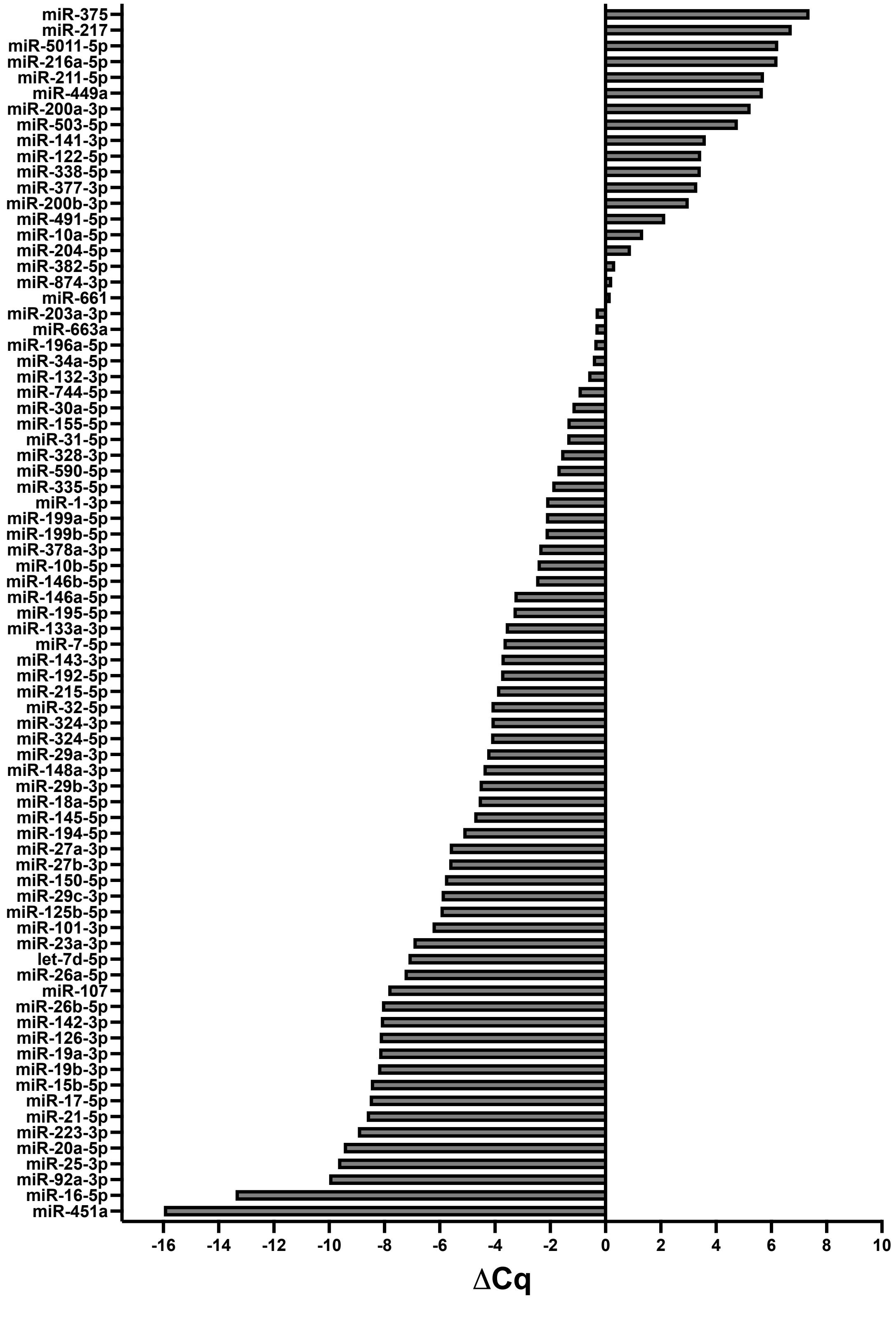

Figure S2. MicroRNA expression profile of the pooled suction device samples (n=7) in Qiagen's fibrosis array. Results are normalized to the mean of six pre-defined housekeeper genes and displayed as ΔCq.
